# Analgesic and Sedative Effect of Fentanyl Versus Dexmedetomidine Infusion in Postoperative Mechanically Ventilated Children After Open Abdominal Surgeries: Randomized Controlled Trial

**DOI:** 10.1155/anrp/9699738

**Published:** 2025-07-03

**Authors:** Amany Mohamed Abotaleb, Mai Rabie Elsheikh, Khalid Mohamed Elshimy, Mohamed Elsaid AbdelFattah

**Affiliations:** ^1^Anesthesiology, Surgical Intensive Care and Pain Medicine, Faculty of Medicine, Tanta University, Tanta, Egypt; ^2^Pediatrics and Neonatology Department, Faculty of Medicine, Tanta University, Tanta, Egypt; ^3^Pediatric Surgery Department, Faculty of Medicine, Tanta University, Tanta, Egypt; ^4^Anesthesiology, Surgical Intensive Care and Pain Medicine, National Cancer Institute, Cairo, Egypt

**Keywords:** dexmedetomidine, fentanyl, mechanical ventilation, pediatric, postoperative, sedation

## Abstract

**Background:** Optimal sedation and analgesia management in mechanically ventilated (MV) children post-abdominal surgery remain controversial. This study compared the efficacy and safety of fentanyl versus dexmedetomidine infusion in this population.

**Methods:** A randomized, double-blinded study enrolled 54 MV children aged 4–11 years post-open abdominal surgeries. Patients received either fentanyl (1 μg/kg bolus, 1–5 μg/kg/h infusion) in Group F or dexmedetomidine (1 μg/kg bolus, 0.2–0.7 μg/kg/h infusion) in Group D. Hemodynamic parameters, sedation (COMFORT-B scale), pain (FLACC scale), and weaning times were assessed.

**Results:** Group D showed significantly lower mean arterial pressure and heart rates from 6 to 24 h post-intervention (*p* < 0.05). Oxygen saturation remained similar between groups. Dexmedetomidine provided superior sedation (COMFORT-B: 7 [6–8] vs. 8 [7–8], *p*=0.022) and analgesia (FLACC: 1 [1–2.5] vs. 2 [2–3], *p*=0.005). However, dexmedetomidine achieved faster weaning (25.89 ± 2.01 vs. 29.19 ± 1.44 h, *p* < 0.001) and higher extubation times (51.93 ± 4.84 vs. 43.78 ± 5.32 min, *p* < 0.001).

**Conclusions:** While dexmedetomidine offered better sedation and pain control, fentanyl facilitated quicker weaning and extubation from MV and better hemodynamics in postoperative MV children after open abdominal surgeries.

**Trial Registration:** ClinicalTrials.gov identifier: NCT06994273

## 1. Introduction

Proper sedation and analgesia are critical components of postoperative care in pediatric intensive care units (PICUs), particularly for children requiring mechanical ventilation (MV) [1]. Effective sedation and analgesia not only alleviate pain and anxiety but also facilitate aggressive ICU therapies, reduce cellular metabolism, prevent accidental removal of medical devices, and enhance patient comfort [2].

Despite their importance, the choice of sedative and analgesic agents in PICUs often relies on clinician experience and estimated patient needs, which can lead to variability in outcomes [3]. Fentanyl and dexmedetomidine are two commonly used agents for sedation and analgesia in PICUs, each with distinct advantages and potential drawbacks. Fentanyl, a potent opioid, is widely used for its rapid pain relief and ability to reduce pain-related behaviors and heart rate (HR) [4]. However, its use can be associated with side effects such as respiratory depression and tolerance, which may prolong MV dependency [5].

Dexmedetomidine, a highly selective alpha-2 adrenergic agonist, offers sedative and analgesic benefits with a lower risk of respiratory depression, making it an attractive alternative for pediatric sedation [6]. It has been studied extensively in various ICU settings, including as a primary sedative, a bridge to extubation, and a treatment for ICU delirium [7].

For children undergoing abdominal surgeries, the choice between these agents is particularly relevant due to the need to balance adequate pain control and sedation with early respiratory recovery and mobilization.

This study aims to compare the safety and efficacy of fentanyl and dexmedetomidine infusions in postoperative MV children following open abdominal surgeries.

## 2. Materials and Methods

A randomized double-blinded study was conducted at the Pediatric and Surgical ICU of Tanta University Hospital and National Cancer Institute, Egypt, from April 2023 to October 2024. Fifty-four MV children aged 4–11 years who required a minimum of 24 h of MV following open abdominal surgeries were enrolled. This study was done after approval from the Ethical Committee of the Faculty of Medicine, Tanta University, Tanta, Egypt (36264PR315/8/23). The study was conducted in accordance with the Declaration of Helsinki. Written informed consent was obtained from all participants' parents or guardians.

Patients with significant congenital anomalies, chromosomal abnormalities, neurologic conditions prohibiting sedation evaluation, neuromuscular diseases, or those receiving neuromuscular blockers were excluded.

### 2.1. Randomization and Blindness

Participants were randomly assigned to two equal groups using computer-generated random numbers (using https://www.randomizer.org/) in sealed opaque envelopes. The fentanyl group received fentanyl as a 1 μg/kg bolus over 10 min, followed by a 1–5 μg/kg/h intravenous infusion after 10–15 min. Dexmedetomidine group received dexmedetomidine as a 1 μg/kg bolus over 10 min, followed by a 0.2–0.7 μg/kg/h intravenous infusion after 10–15 min.

All PICU staff, including nurses and pharmacists, were blinded to the treatment allocation to ensure unbiased assessment and management of the patients.

All participants underwent a comprehensive clinical examination and detailed history-taking. Daily investigations included complete blood count, liver function tests, blood urea nitrogen, creatinine, and arterial blood gases.

Noninvasive blood pressure, HR, and oxygen saturation were continuously monitored and recorded at baseline and 2, 5, 10, 15, and 30 min and 2, 6, 12, 24, and 48 h post-infusion initiation.

Ventilatory parameters, including respiratory rate, peak inspiratory pressure, and FiO_2_, were monitored at baseline and 6, 12, 24, and 48 h post-infusion under assisted pressure-controlled ventilation mode.

Assent was not obtained from the patients due to their MV status and young age.

The COMFORT-B scale [8] assessed sedation levels, with 6–10 indicating excessive sedation, 11–22 indicating adequate sedation, and > 23 indicating insufficient sedation. Pain was evaluated using the FLACC scale [9], with scores of 0 indicating no pain, 1–3 mild, 4–6 moderate, and 7–10 severe pain or discomfort.

Infusion rates were adjusted to achieve a COMFORT-B score of 11–22 for sedation and a FLACC score < 3 for analgesia. Rescue analgesia (fentanyl 0.5 μg/kg IV) was administered for FLACC scores ≥ 4, while rescue sedation (midazolam 50–150 μg/kg IV) was given for COMFORT-B scores ≥ 23. Midazolam was selected for rescue sedation due to its rapid onset and short duration of action, which is suitable for managing acute agitation or insufficient sedation. Dexmedetomidine was not used as a rescue medication to avoid confounding the study results. The number of additional sedative or analgesic doses required was recorded for both groups.

The time from ventilator weaning eligibility and time from eligibility to extubation were monitored until discharge. Eligibility for ventilator weaning was determined based on standard clinical criteria, including stable hemodynamics (mean arterial pressure within the normal range for age), adequate oxygenation (SpO_2_ > 92% on FiO_2_ ≤ 0.4), and spontaneous breathing efforts.

Weaning and extubation were conducted based on the clinician judgment.

The primary outcomes included effects on patient hemodynamics, weaning and extubation times, and the number of additional sedative or analgesic doses required. Secondary outcomes encompassed any recorded complications.

### 2.2. Sample Size Calculation

The sample size calculation was done by G∗Power 3.1.9.2 (Universitat Kiel, Germany). We performed a pilot study (five cases in each group), and we found that the mean (±SD) time to eligibility for weaning was 12.2 ± 8.04 h in the fentanyl group and 7.2 ± 2.77 h in the dexmedetomidine group. The sample size was based on the following considerations: 0.83 effect size, 95% confidence limit, 80% power of the study, and group ratio 1:1, and three cases were added to each group to overcome dropout. Therefore, we recruited 27 patients in each group.

### 2.3. Statistical Analysis

Statistical analysis was conducted using IBM SPSS v27 (Armonk, NY, USA). The Shapiro–Wilk test and histograms assessed data normality. Parametric quantitative data were given as mean and SD and examined using an unpaired Student's *t*-test. The median and IQR were used to examine quantitative nonparametric data using the Mann–Whitney test. When appropriate, the chi-square test or Fisher's exact test was used to examine qualitative variables as frequency (%). A two-tailed *p* value < 0.05 indicated significance.

## 3. Results

**Figure 1:**
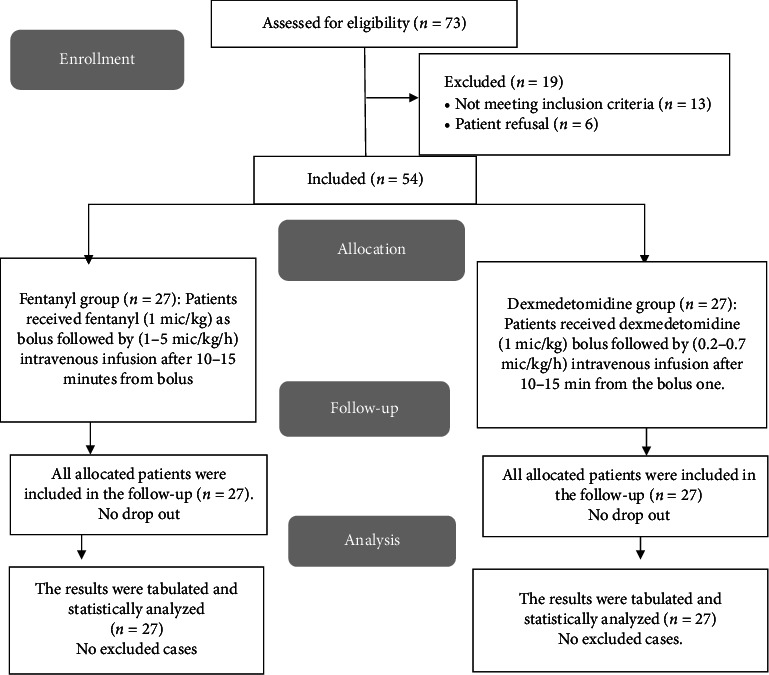
CONSORT flowchart of the enrolled patients.

About 73 children were evaluated for this investigation. 13 patients did not qualify, and six guardians declined participation. Two random groups of 27 patients were formed from the remaining 54 patients. All assigned patients were followed up and statistically analyzed (Figure 1).

Patient characteristics, type, and surgery indication are presented in Table 1.

Both groups had similar MAP and HR measurements at various time points; however, the dexmedetomidine group had significantly lower values at 6, 12, and 24 h than the fentanyl group (*p* < 0.05) (Table 2).

**Figure 2:**
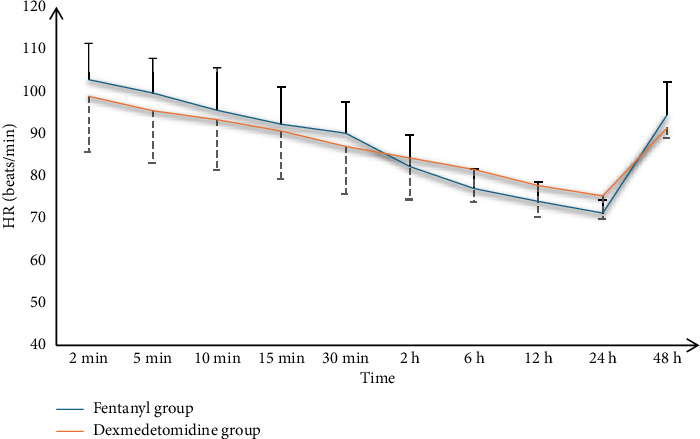
O_2_ saturation of the studied groups.

Both groups' O_2_ saturation values at 2, 5, 10, 15, and 30 min and 2, 6, 12, 24, and 48 h were insignificant (Figure 2).

Dexmedetomidine significantly decreased weaning time compared to fentanyl and increased extubation times compared to fentanyl (*p* < 0.001). PICU length of stay was insignificantly different between both groups. Duration of MV was significantly higher in the dexmedetomidine group than in the fentanyl group (*p* < 0.001). Mortality did not occur in any patient in both groups (Table 3).

**Figure 3:**
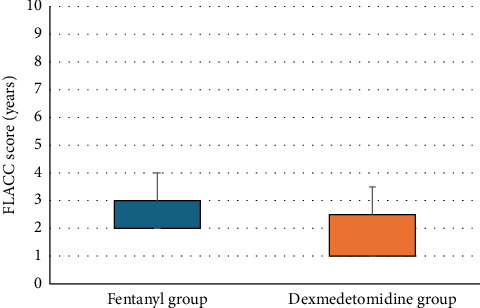
FLACC score of the studied groups.

Dexmedetomidine significantly reduced COMFORT-B, FLACC scores, and rescue analgesia consumption compared to fentanyl (*p* < 0.05) (Table 4 and Figure 3).

## 4. Discussion

The optimal management of sedation and analgesia in MV children following surgeries remains a subject of ongoing debate in PICUs, with recent studies exploring the comparative efficacy of various pharmacological agents like fentanyl and dexmedetomidine [10, 11].

Our study revealed exciting patterns in the hemodynamic responses to fentanyl and dexmedetomidine. Initially, MAP and HR were similar in groups until 6 h post-intervention, after which Group D showed consistently lower MAP and HR than Group F, a trend that persisted through 24 h.

Prasad et al.'s [10] study in pediatric cardiac patients showed comparable hemodynamic parameters between the two drugs, with only modest HR decreases in the dexmedetomidine group, while Sun et al.'s [12] research demonstrated stable hemodynamics with dexmedetomidine across multiple dosage levels, except for a transient MAP elevation at higher doses in children undergoing laparoscopic hernia repair. While our study found statistically significant differences in hemodynamic parameters between groups at 6-24 hours, these parameters remained within age-appropriate physiological ranges for pediatric patients. None of the patients required intervention for these hemodynamic changes, suggesting these differences may not be clinically meaningful in practice.

One of the most striking findings in our study was the difference in weaning and extubation times between both groups (D and F). Group D achieved eligibility for weaning significantly earlier than Group F (25.89 ± 2.01 h vs. 29.19 ± 1.44 h, *p* < 0.001). Furthermore, once eligibility for weaning was achieved, Group D also exhibited a longer time to extubation than Group F (51.93 ± 4.84 min vs. 43.78 ± 5.32 min, *p* < 0.001).

These findings contradict several previous research studies. For instance, Saleh [13] found that the average time needed for extubation when infusion stopped was significantly shorter in Group D (136.2 ± 54.2 min) than in Group F (341.4 ± 125.4 min). Similarly, Zedan et al. [14] found that compared to a placebo group, dexmedetomidine dramatically shortened the time it took to extubate MV preterm infants.

The discrepancy between our findings and these studies might be linked to several factors, including variations among patients like age, surgical procedures, dosing regimens, or institutional weaning protocols.

Our study revealed that Group D demonstrated lower scores on the COMFORT-B scale than Group F (7 [6–8] vs. 8 [7, 8], *p*=0.022), indicating potentially better sedation. Similarly, the FLACC scale scores were significantly lower in Group D (1 [1–2.5] vs. 2 [2, 3], *p*=0.005), suggesting superior pain control.

These findings align with several other studies in the literature. El Shamaa and Ibrahim [15] reported that caudal dexmedetomidine added to bupivacaine provided significantly longer periods of analgesia and sedation than morphine in pediatric patients undergoing lower abdominal and perineal surgeries. Fares et al. [16] found that intrathecal dexmedetomidine resulted in lower FLACC scores at 6, 8, and 12 h postoperatively than fentanyl in pediatrics with malignancies embarking on a significant abdominal operation.

While the differences in COMFORT-B scores were statistically significant, both groups maintained scores within acceptable clinical ranges (COMFORT-B < 10 indicating adequate sedation and FLACC < 3 indicating mild pain). Dexmedetomidine significantly reduced rescue analgesia consumption compared to fentanyl.

Observed evidence indicates dexmedetomidine's superiority over fentanyl in accelerating post-anesthetic recovery (5–10 min differential) and mitigating emergence agitation, thereby enhancing postsurgical recovery courses [17]. These advantages stem from dexmedetomidine's distinct method of action as an agonist for the *α*2-adrenergic receptor, providing sedation and analgesia without significant respiratory depression [18, 19], making it a superior choice for accelerating post-anesthetic recovery compared to fentanyl.

While our study did not specifically assess the return of abdominal function after surgery, previous research in adults has suggested that dexmedetomidine may have beneficial effects on the recovery of gastrointestinal function following abdominal surgery [20]. This potential benefit warrants further investigation in pediatric populations as a future direction.

Single-center design and small sample size could limit the generalizability of this study. Long-term follow-up was not conducted, precluding assessment of any delayed effects or complications associated with either sedation regimen. Additionally, the study only examined one type of sedation protocol at a fixed dose without exploring different drug combinations or dosing regimens that might yield varying outcomes. The weaning and extubation decisions were left to the discretion of the clinicians, which may have introduced variability in these processes. Additionally, since patients in the dexmedetomidine group received fewer bolus sedation doses compared to those in the fentanyl group, it is possible that clinicians perceived them as less ready for extubation. This could have influenced the timing of extubation and weaning between the two groups. These factors should be considered when interpreting the results, as they may have impacted the observed differences in weaning and extubation times.

## 5. Conclusions

In postoperative MV children after open abdominal surgeries, dexmedetomidine provided better sedation and pain control, as evidenced by lower COMFORT-B and FLACC scores. However, Group F achieved faster weaning and extubation times and better hemodynamics. These findings suggest that while dexmedetomidine offers superior comfort, fentanyl may facilitate quicker recovery from MV in this patient population.

## Figures and Tables

**Table 1:** Patient characteristics, type, and surgery indication of the studied groups.

	Fentanyl group (*n* = 27)	Dexmedetomidine group (*n* = 27)
Age (years)	6.85 ± 1.35	6.48 ± 1.34
Sex		
Male	19 (70.37%)	16 (59.26%)
Female	8 (29.63%)	11 (40.74%)
Weight (kg)	29.59 ± 5.06	27.3 ± 5.73
Height (cm)	123.02 ± 8.87	118.41 ± 8.91
BMI (kg/m^2^)	19.47 ± 1.53	19.27 ± 2.65
Types of surgery		
Appendectomy	4 (14.8%)	3 (11.11%)
Bowel resection	1 (3.7%)	2 (7.4%)
Colectomy	5 (18.5%)	4 (14.8%)
Gastrectomy	6 (22.2%)	9 (33.3%)
Open cholecystectomy	8 (29.6%)	7 (25.9%)
Splenectomy	3 (11.11%)	2 (7.4%)
Indication		
Acute abdominal pain	17 (62.9%)	15 (55.5%)
Appendicitis	5 (18.5%)	3 (11.11%)
Cholecystitis	13 (48.1%)	12 (44.4%)
Cancer	14 (51.8%)	18 (66.6%)
Obstruction of the bowel	2 (7.4%)	2 (7.4%)

*Note:* Data are presented as mean ± SD or frequency (%).

Abbreviation: BMI, body mass index.

**Table 2:** Mean arterial blood pressure and heart rate of the studied groups.

	Fentanyl group (*n* = 27)	Dexmedetomidine group (*n* = 27)	*p* value
*Mean arterial blood pressure (mmHg)*			
2 min	84.19 ± 5.97	82.67 ± 7.83	0.427
5 min	81.96 ± 6.17	80.67 ± 6.84	0.468
10 min	79.52 ± 6	78.81 ± 3.62	0.604
15 min	78.33 ± 7.06	76.96 ± 2.79	0.353
30 min	76.63 ± 5.68	74.56 ± 3.27	0.106
2 h	72.56 ± 6.17	70.59 ± 3.73	0.163
6 h	70.41 ± 5.68	65.7 ± 4.3	**0.001^∗^**
12 h	67.93 ± 6.34	63.37 ± 3.78	**0.002^∗^**
24 h	65.41 ± 5.57	61.11 ± 3.31	**0.001^∗^**
48 h	86.59 ± 5.26	84.44 ± 2.89	0.068

*Heart rate (beats/min)*			
2 min	102.81 ± 8.56	98.89 ± 13.09	0.198
5 min	99.63 ± 8.19	95.48 ± 12.33	0.151
10 min	95.56 ± 10.07	93.33 ± 11.87	0.462
15 min	92.26 ± 8.92	90.63 ± 11.25	0.558
30 min	90.15 ± 7.38	87 ± 11.13	0.226
2 h	82.81 ± 7.63	79.22 ± 8.56	0.110
6 h	81.04 ± 6.63	76.33 ± 7.97	**0.022^∗^**
12 h	77.44 ± 4.55	72.74 ± 6.96	**0.005^∗^**
24 h	75.26 ± 3.73	72.81 ± 5.02	**0.047^∗^**
48 h	94.52 ± 7.81	91.41 ± 2.37	0.052

*Note:* Data are presented as mean ± SD. Bold denotes statistical significance.

^∗^Significant as *p* value ≤ 0.05.

**Table 3:** Time to eligibility for weaning, time from eligibility for weaning to extubation, PICU length of stay, duration of mechanical ventilation, and mortality of the studied groups.

	Fentanyl group (*n* = 27)	Dexmedetomidine group (*n* = 27)	*p* value
Time to eligibility for weaning (h)	29.19 ± 1.44	25.89 ± 2.01	**< 0.001^∗^**
Time from eligibility for weaning to extubation (min)	43.78 ± 5.32	51.93 ± 4.84	**< 0.001^∗^**
PICU length of stay	4.41 ± 1.05	4.89 ± 1.12	0.109
Duration of mechanical ventilation (days)	18.19 ± 7.11	29.37 ± 12.44	**< 0.001^∗^**
Mortality	0 (0%)	0 (0%)	**—**

*Note:* Data are presented as mean ± SD. Bold denotes statistical significance.

^∗^Significant as *p* value ≤ 0.05.

**Table 4:** COMFORT-B scale, FLACC scale, and rescue analgesia consumption of the studied groups.

	Fentanyl group (*n* = 27)	Dexmedetomidine group (*n* = 27)	*p* value
COMFORT-B scale (infusion rate)	8 (7-8)	7 (6–8)	**0.022^∗^**
FLACC scale	2 (2-3)	1 (1–2.5)	**0.005^∗^**
Rescue analgesia consumption	6.44 ± 2.15	4.67 ± 1.52	**< 0.001^∗^**

*Note:* Data are presented as median (IQR). Bold denotes statistical significance.

Abbreviation: FLACC: face, legs, activity, cry, and consolability.

^∗^Significant as *p* value ≤ 0.05.

## Data Availability

The data that support the findings of this study are available from the corresponding author upon reasonable request.
